# Mucosal healing assessment in Crohn’s disease with normalized iodine concentration from dual-energy CT enterography: comparison with endoscopy

**DOI:** 10.1186/s13244-023-01397-7

**Published:** 2023-04-13

**Authors:** Chao Zhu, Jing Hu, Chang Rong, Jianhua Zhou, Bo Zhang, Yankun Gao, Cuiping Li, Jianying Li, Xingwang Wu

**Affiliations:** 1 Department of Radiology, The First Affiliated Hospital of Anhui Medical University, No. 218 Jixi Road, Shushan District, Hefei, 230022 Anhui Province People’s Republic of China; 2 Department of Gastroenterology, The First Affiliated Hospital of Anhui Medical University, Hefei, People’s Republic of China; 3 CT Research Center, GE Healthcare China, Shanghai, People’s Republic of China

**Keywords:** Crohn’s disease, Iodine, Tomography (X-ray computed), Infliximab, Colonoscopy

## Abstract

**Objectives:**

Mucosal healing (MH) is an important goal in the treatment of patients with Crohn’s disease (CD). Noninvasive assessment of MH with normalized iodine concentration (NIC) is unknown.

**Methods:**

In this retrospective study, 94 patients with diagnosed CD underwent endoscopy and dual-energy CT enterography (DECTE) at the post-infliximab treatment review. Two radiologists reviewed DECTE images by consensus for assessing diseased bowel segments of the colon or terminal ileum, and the NIC was measured. Patients were divided into transmural healing (TH), MH and non-MH groups. The diagnostic performance of the MH and non-MH groups with clinical factors and NIC was assessed utilizing receiver operating characteristic (ROC) curve analysis.

**Results:**

Of the 94 patients included in our study, 8 patients achieved TH, 34 patients achieved MH, and 52 patients did not achieve MH at the post-IFX treatment review. The area under the ROC curve (AUC), sensitivity, specificity, and accuracy values were 0.929 (95% confidence interval [CI] 0.883–0.967), 0.853, 0.827, and 0.837, respectively, for differentiating MHs from non-MHs, and the optimal NIC threshold was 0.448. The AUC of the combined model for distinguishing MHs from non-MHs in CD patients, which was based on the NIC and calprotectin, was 0.964 (95% CI 0.935–0.987).

**Conclusions:**

The normalized iodine concentration measurement in DECTE has good performance in assessment MH in patients with CD. Iodine concentration from DECTE can be used as a radiologic marker for MH.

## Key points

1. IFX treatment has 45% efficiency for achieving MH in our study.
2. The AUC of the NIC model for assessment of MH was 0.929.
3. NIC from DECTE can be used as a radiologic marker for MH.

## Introduction

Mucosal healing (MH) is currently the long-term treatment goal for Crohn's disease (CD) [1–3]. Achieving transmural healing (TH) and MH in patients with CD can reduce hospitalization and surgery rates and maintain long-term remission of clinical symptoms [4–6]. Accurate determination of TH and MH can help to assess the prognosis of patients with CD and to personalize treatment [7, 8]. Currently, the evaluation of MH relies on endoscopy, but it is invasive and unsuitable for patients with intestinal strictures, perforated bowel walls, or intestinal fistulas. It is reported that endoscopy can miss affected segments reportedly in 30.6% [9]. Consequently, non-invasive assessment of MH is of increasing interest to scholars and clinicians [10].

Cross-sectional imaging is crucial in the assessment of CD [11]. However, some of the bowel segments that have achieved MH endoscopically continue to show wall thickening (> 3 mm) on cross-sectional images such as magnetic resonance enterography (MRE) or computed tomography enterography (CTE) images. This suggests that there may be ongoing inflammation of the intestinal wall [12, 13]. Dual-energy CT enterography (DECTE) allows quantitative studies of the iodine concentration of diseased tissue, which has been shown to be a substitute marker for perfusion in various abdominal imaging applications [14, 15]. Iodine concentration obtained from specific intestinal regions identified by DECTE has been suggested as a marker of CD activity and histopathologic analysis [16, 17]. However, to our knowledge, there are no reports on DECTE-based assessment of MH in CD patients. The aim of this study was to assess whether the normalized iodine concentration (NIC) of the thickened wall of the terminal ileal or colonic segment could be used to diagnose endoscopic MH in CD patients.

## Materials and methods

### Study design and patients

This retrospective study received approval from the Clinical Medical Research Ethics Committee of our hospital. The informed consent requirement was waived.

The inclusion criteria were as follows: (1) patients with confirmed active CD at baseline; (2) patients with ulcerative inflammation in the colon or terminal ileum at baseline.

The exclusion criteria were as follows: (1) no DECTE and endoscopy examination after infliximab (IFX) treatment; and (2) poor quality of DECTE images for analysis.

**Fig. 1:**
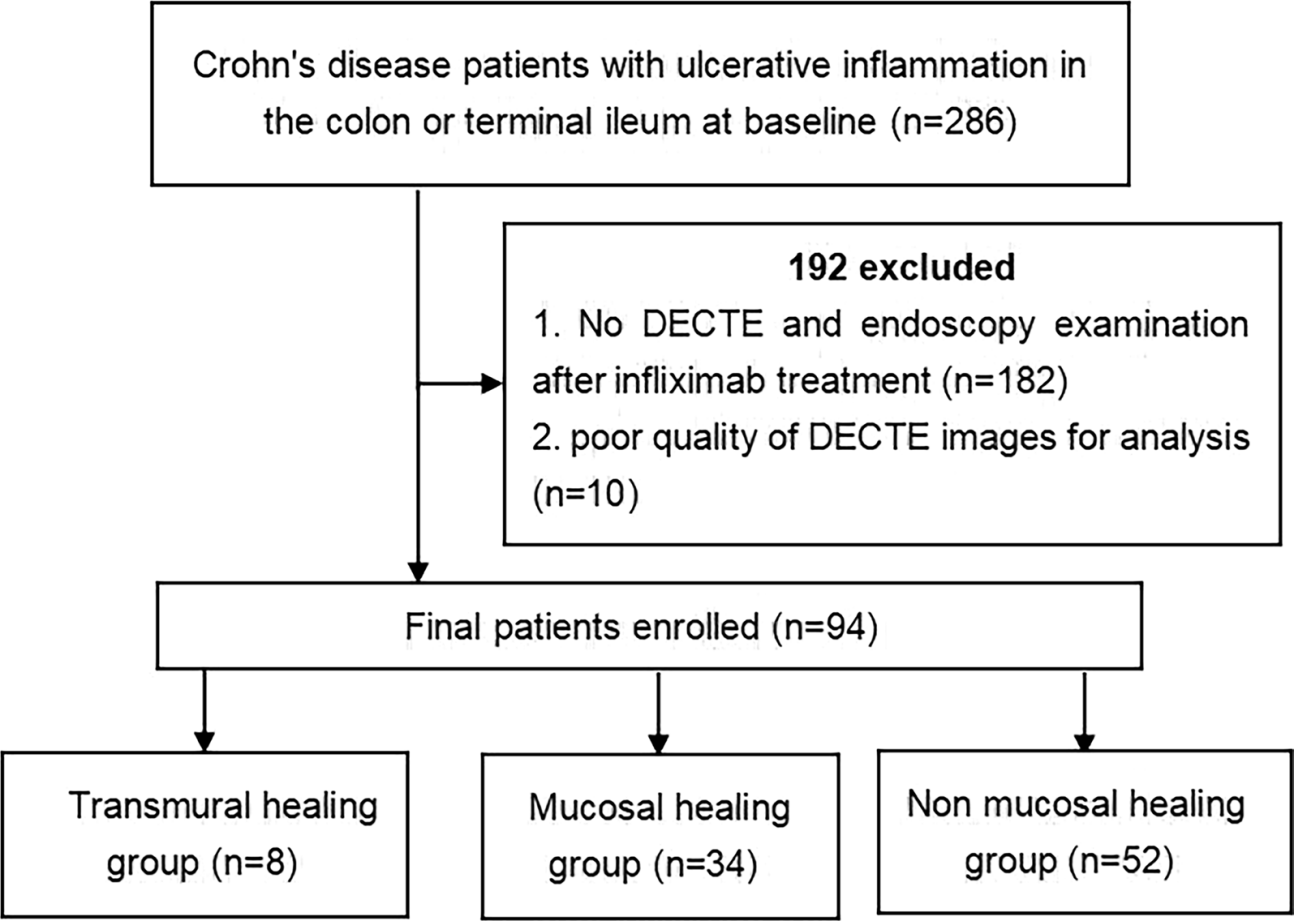
The flow diagram of the study

The study flow diagram is shown in Fig. 1. The diagnosis of CD is based on a combination of clinical, endoscopic, stool, biochemical, and histological investigations [18]. The following data were collected at the post-IFX treatment review: age, sex, disease duration, calprotectin, C-reactive protein (CRP) level, serum albumin level, white blood cell (WBC) count, simple endoscopic score for CD, and Harvey Bradshaw Index (HBI).

Each endoscopy was performed by two gastroenterologists with more than 15 years of colonoscopy experience. One of the primary operators performed the colonoscopy and the other assisted the primary operator in viewing the colonoscopy images, and evaluated each patient for ulcerative inflammation at baseline and for MH at the post-IFX treatment review. The MH was evaluated using the Simple Endoscopic Score for CD (SES-CD) in colonic and terminal ileum lesions. MH was characterized as an SES-CD < 3 with the absence of mucosal ulcers after IFX treatment in CD patients who had ulcerative inflammation at baseline [19–21].

### CT scanning

DECTE was performed on all included individuals at the post-IFX treatment review. Before the examination, all patients received standardized intestinal preparation. Patients were given a light diet and bowel preparation the day before the examination. Approximately 45 min before the CT examination, each patient drank 1500–2000 ml of 2.5% isotonic mannitol solution. DECTE was performed using a GE Revolution CT scanner equipped with spectral imaging mode (GSI Assist, GE Healthcare). The scan protocol for DECTE was as follows: fast tube voltage switching between 80 and 140 kVp; tube current, 350 mA; pitch, 0.984:1; rotation time, 0.75 s; and reconstructed layer thickness, 1.25 mm. A contrast agent (iohexol, 320 mg/mL) was administered using a high-pressure syringe at 1.5 mL/kg bodyweight via peripheral veins at a rate of 3.0 mL/s. The enteric and venous phases of DECTE were collected at 45 s and 70 s after contrast injection, respectively. On a Gemstone Spectral Imaging (GSI) viewer workstation (AW4.7, GE Healthcare), the dual-energy image sets were rebuilt. The GSI viewer quantified iodine concentration by reviewing images of the decomposition spectra of water and iodine-based materials.

### Quantitative iodine concentration analysis

Two radiologists with more than 6 and 20 years of expertise in diagnostic abdominal imaging reviewed DECTE enteric phase images by consensus for assessment of the diseased bowel segment of the colon or terminal ileum. TH was defined as wall thickness < 3 mm with the absence of ulcers, edema, enhancement, and complications for all ileocolonic segments [1].

The selection of the diseased bowel segment for quantitative iodine concentration analysis was based on the following criteria: (1) a segment of the colon or terminal ileum; (2) for MH patients, selection of the diseased bowel segment achieved MH; (3) for non-MH patients, selection of the diseased bowel segment with the ulcerative inflammation; (4) thickening (> 3 mm) of the diseased bowel segment wall with the highest enhancement [17]. On the GSI viewer workstation, a circular region of interest (ROI) (area, 5 mm × 5 mm) was placed on the thickened wall in the area of the highest iodine concentration for quantitative analysis. Areas of intraluminal air or fluid and perienteric fat were carefully avoided. All measurements were performed three times at various image levels of the same bowel segment to ensure consistency, and the average values were computed. The iodine concentration in the diseased intestinal wall was derived from the iodine-based material decomposition images. To reduce patient and scan time variations, the iodine concentration measurement of the lesion was normalized to that of the aorta or iliac artery by a circular ROI (area, 5 mm × 5 mm) at the same level on the iodine map to obtain a normalized iodine concentration (NIC) [22].

### Construction of the combined model

Univariate analysis was used to compare the differences in clinical factors and NIC collected at the post-IFX treatment between the with and without MH patient groups. The significant variables (*p* < 0.05) acquired in the univariate analysis were employed as inputs. Multivariate logistic regression analysis (*p* < 0.05) and variance inflation factors were used to build a combined model. A combined model was constructed by combining the significant variables of clinical factors and the NIC. A receiver operating characteristic (ROC) curve analysis was used to assess the diagnostic performance for discriminating between the MH and non-MH groups.

### Statistical analysis

Continuous variables were presented as the mean ± standard deviation when they followed a normal distribution and were compared using the independent sample *t test*; otherwise, they were presented as the median (P25, P75) and tested using the Mann‒Whitney *U* test. The Fisher’s exact test or chi-square test was used for categorical variables. Pearson’s correlation test was used to analyze inter-reader agreement for the iodine quantification by two radiologists. A two-sided *p* < 0.05 was considered significant. The above statistical analyses were performed using R 3.5.1. (R Institute for Statistical Computing).

## Results

### Patient characteristics

A total of 286 CD patients from November 2016 to December 2021 were enrolled in this study. The study ultimately included 94 patients who met the inclusion and exclusion criteria. Of the 94 patients included in our study (Fig. 1), 8 patients achieved TH, 34 patients achieved MH, and 52 patients did not achieve MH at the post-IFX treatment review. Clinical characteristics of patients with or without MH and TH are shown in Tables 1 and 2. Between the MH and non-MH groups, there was no significant difference in terms of patient age, sex, HBI, or perianal disease (*p* > 0.05). Calprotectin was significantly lower in the MH group (78.8 ug/g) than in the non-MH group (931.32 ug/g) (*p* < 0.001). CRP was significantly lower in the MH group (0.73 mg/dL) than in the non-MH group (7.74 mg/dL) (*p* < 0.001). Serum albumin was significantly higher in the MH group (42.1 g/dL) than in the non-MH group (38.9 g/dL) (*p* < 0.001). Disease duration and WBC were significantly lower in the MH group than in the non-MH group (*p* = 0.009, 0.007).

**Table 1:** Clinical characteristics of patients with or without MH

Characteristics	MH (*n* = 34)	Non-MH (*n* = 52)	*p* value
Gender, *n* (%)			0.852
Female	9 (10.5%)	16 (18.6%)	
Male	25 (29.1%)	36 (41.9%)	
Age (years), median (IQR)	26.5 (21.5, 32.5)	27 (21.75, 33)	0.944
Perianal disease, *n* (%)	19 (22.1%)	25 (29.1%)	0.626
Disease duration (months), median (IQR)	12 (12, 24)	30 (12, 51)	0.009
Calprotectin (mg/kg), median (IQR)	78.81 (18.08, 279.16)	931.32 (408.19, 1329.3)	< 0.001
CRP (mg/dL), median (IQR)	0.73 (0.42, 2.58)	7.74 (3.74, 16.79)	< 0.001
Serum albumin (g/dL), median (IQR)	42.1 (40.92, 43.98)	38.9 (37, 41.3)	< 0.001
HBI, median (IQR)	1.5 (1, 2.75)	2 (1, 4.25)	0.221
WBC (× 10^9^ cells per L), mean ± SD	5.61 ± 1.46	6.55 ± 1.59	0.007

*MH* Mucosal healing, *CRP* C-reactive protein, *HBI* Harvey–Bradshaw index, *WBC* White blood cell, *IQR* Interquartile range, *SD* Standard deviation

**Table 2:** Clinical characteristics of patients with or without TH

Characteristics	TH (*n* = 8)	Non-TH (*n* = 86)	*p* value
Gender, *n* (%)			0.105
Female	5 (5.3%)	25 (26.6%)	
Male	3 (3.2%)	61 (64.9%)	
Perianal disease, *n* (%)	6 (6.4%)	44 (46.8%)	0.276
Age (years), median (IQR)	27 (23.25, 35.25)	27 (21.25, 33)	0.802
HBI, median (IQR)	0 (0, 0.25)	2 (1, 3)	< 0.001
Disease duration (months), median (IQR)	19.5 (12, 36)	24 (12, 48)	0.655
Calprotectin (mg/kg), median (IQR)	86.45 (41.13, 345.91)	401.26 (90.04, 1049.22)	0.207
CRP (mg/dL), median (IQR)	0.88 (0.71, 1.52)	4.21 (0.8, 10.29)	0.037
Serum albumin (g/dL), median (IQR)	41.15 (40.85, 42.92)	40.45 (37.65, 43)	0.149
WBC (× 10^9^ cells per L), mean ± SD	6.36 ± 1.87	6.18 ± 1.6	0.761

*TH* Transmural healing, *CRP* C-reactive protein, *HBI* Harvey–Bradshaw index, *WBC* White blood cell, *IQR* Interquartile range, *SD* Standard deviation

### DECTE iodine concentration endoscopic comparison

**Fig. 2:**
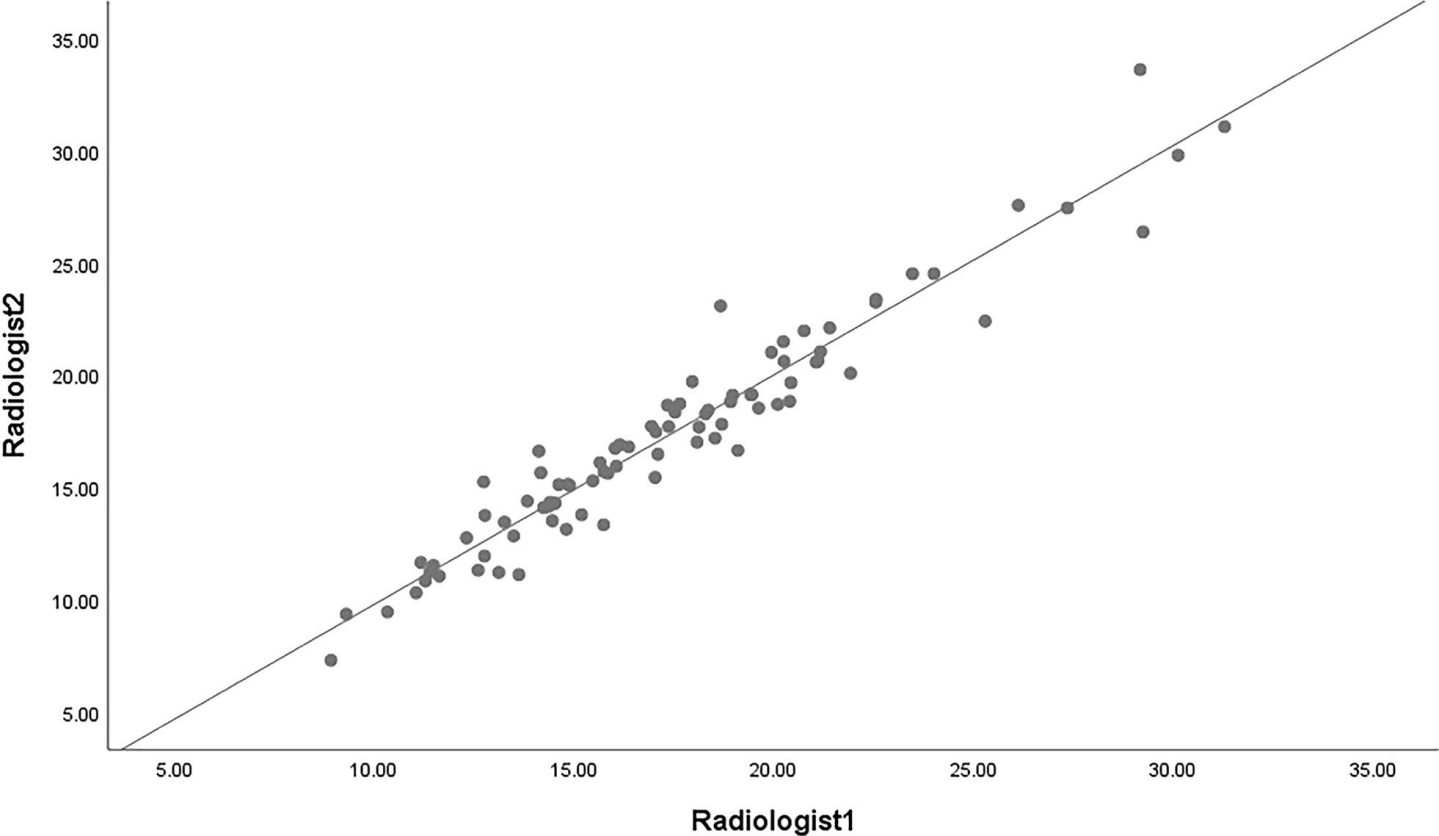
Scatter diagram of iodine quantification by two radiologists

**Fig. 3:**
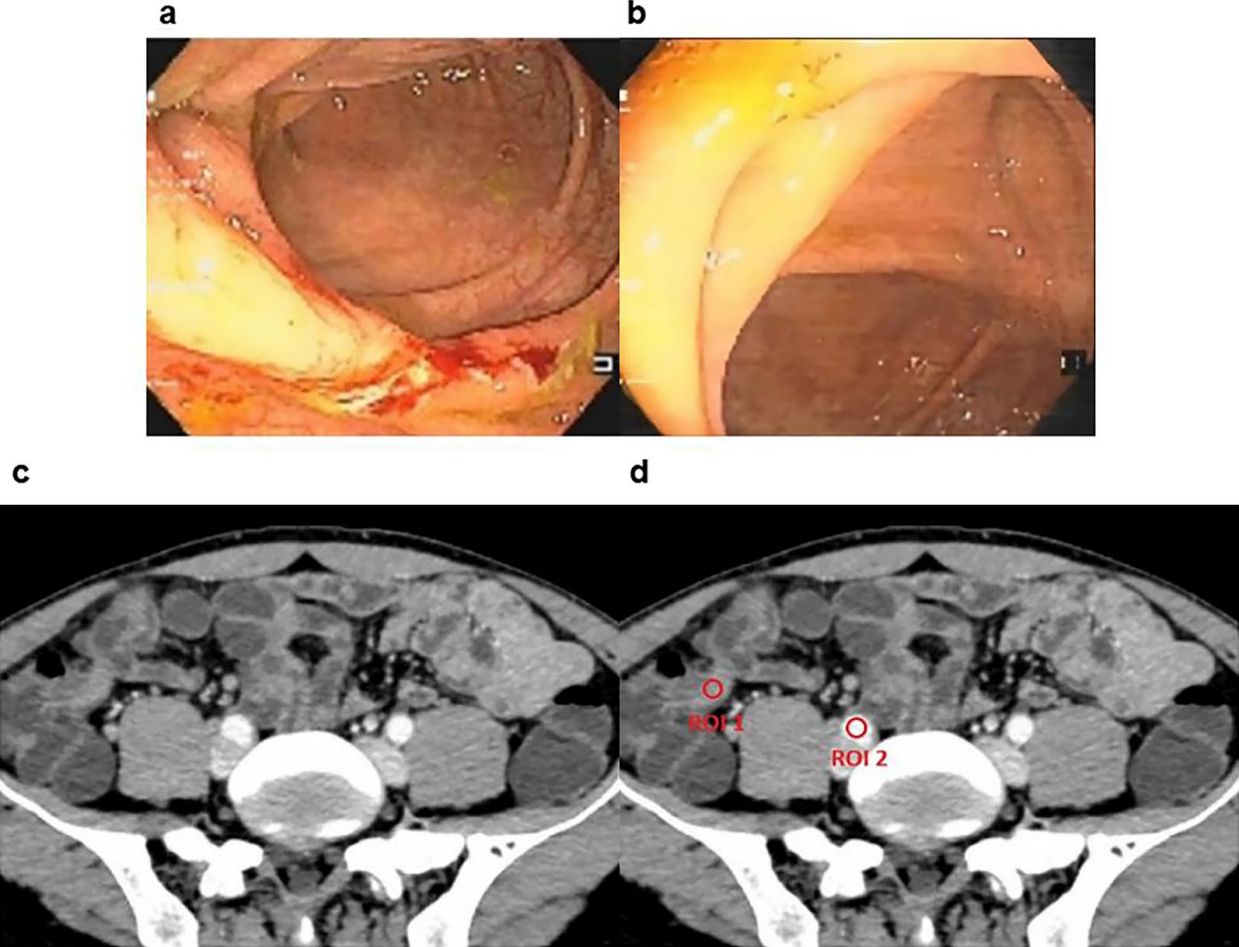
A 39-year-old man with Crohn’s disease achieved endoscopic mucosal healing after 6 months of therapy. **a** Endoscopic images of the terminal ileum show ulcerating inflammation before infliximab treatment. **b** Posttreatment endoscopic images show mucosal healing. **c** On the posttreatment enteric phase axial CTE image, the wall of the terminal ileum is thickened. **d** ROI1 was placed in the thickened wall of the terminal ileum in these iodine-based material decomposition images, and ROI2 was placed in the iliac artery at the same level. The NIC in this thickened wall of the terminal ileum was 0.332

**Fig. 4:**
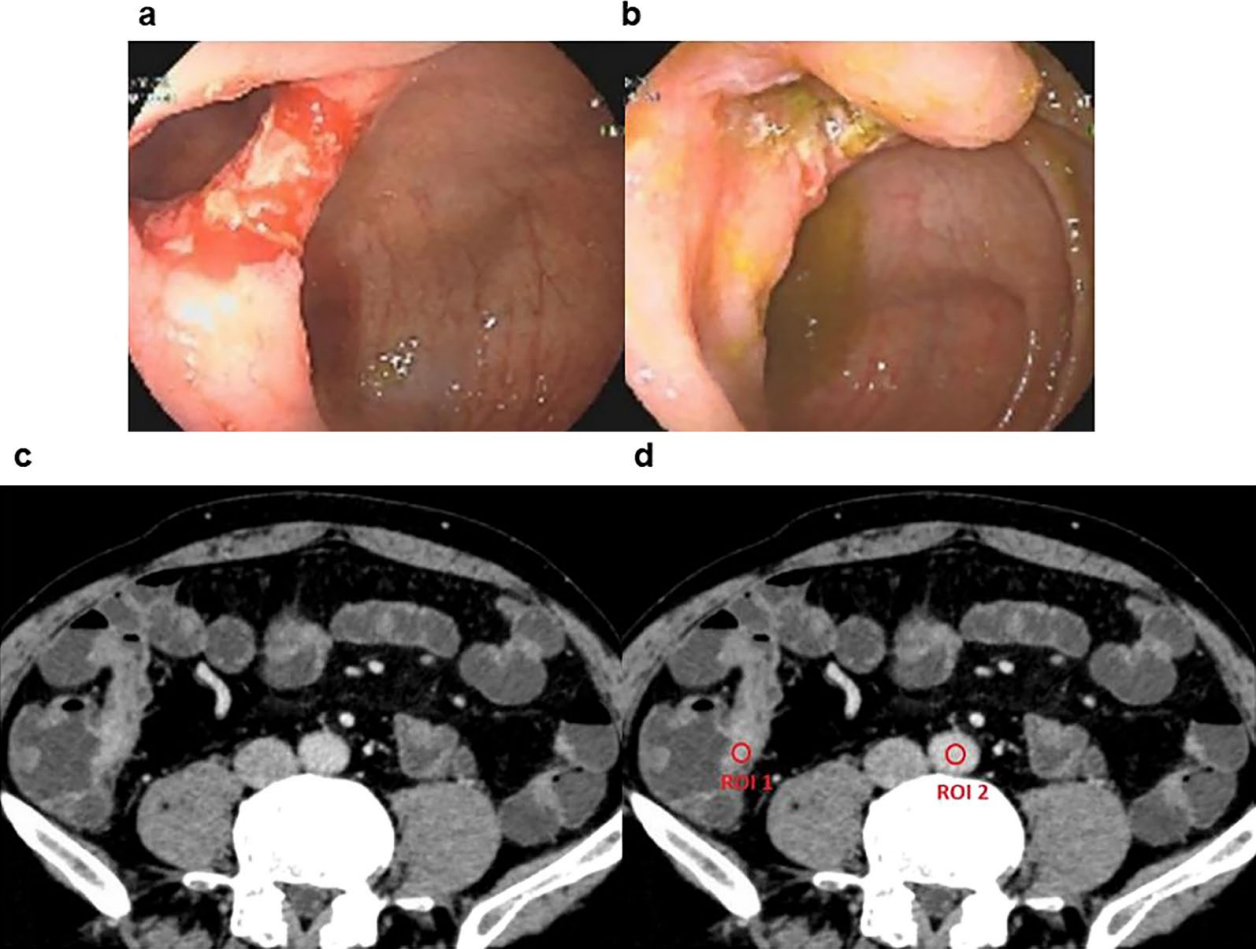
A 52-year-old man with Crohn’s disease did not achieve endoscopic mucosal healing after 8 months of therapy. **a** Endoscopic images of the terminal ileum show ulcerating inflammation before infliximab treatment. **b** Posttreatment endoscopic images still show ulcerating inflammation. **c** On the posttreatment enteric phase axial CTE image, the wall of the terminal ileum is thickened. **d** ROI1 was placed in the thickened wall of the terminal ileum in these iodine-based material decomposition images, and ROI2 was placed in the artery at the same level. The NIC in this thickened wall of the terminal ileum was 0.671

The correlation coefficient of the inter-reader agreement for the iodine quantification was 0.966 (Fig. 2). Segments of the diseased bowel that achieved MH (n = 34, NIC = 0.337 ± 0.012) had a different NIC than segments that had ulcerative inflammation (n = 52, NIC = 0.556 ± 0.016). The NIC and SES-CD of patients with or without MH are shown in Table 3. The area under the ROC curve (AUC), sensitivity, specificity, and accuracy values were 0.929 (95% confidence interval [CI] 0.883–0.967), 0.853, 0.827, and 0.837, respectively. The optimal NIC threshold was 0.448, which could obtain the best combination of sensitivity and specificity, for differentiating MH from non-MH. Representative examples are shown in Figs. 3 and 4.

**Table 3:** NIC and SES-CD of patients with or without MH

Characteristics	MH (*n* = 34)	Non-MH (*n* = 52)	*p* value
NIC, mean ± SD	0.34 ± 0.07	0.56 ± 0.12	< 0.001
SES-CD, median (IQR)	0 (0, 1)	8 (6, 12)	< 0.001

*NIC* Normalized iodine concentration, *SES-CD* Simple endoscopic score for CD

### Establishment of a combined model

**Fig. 5:**
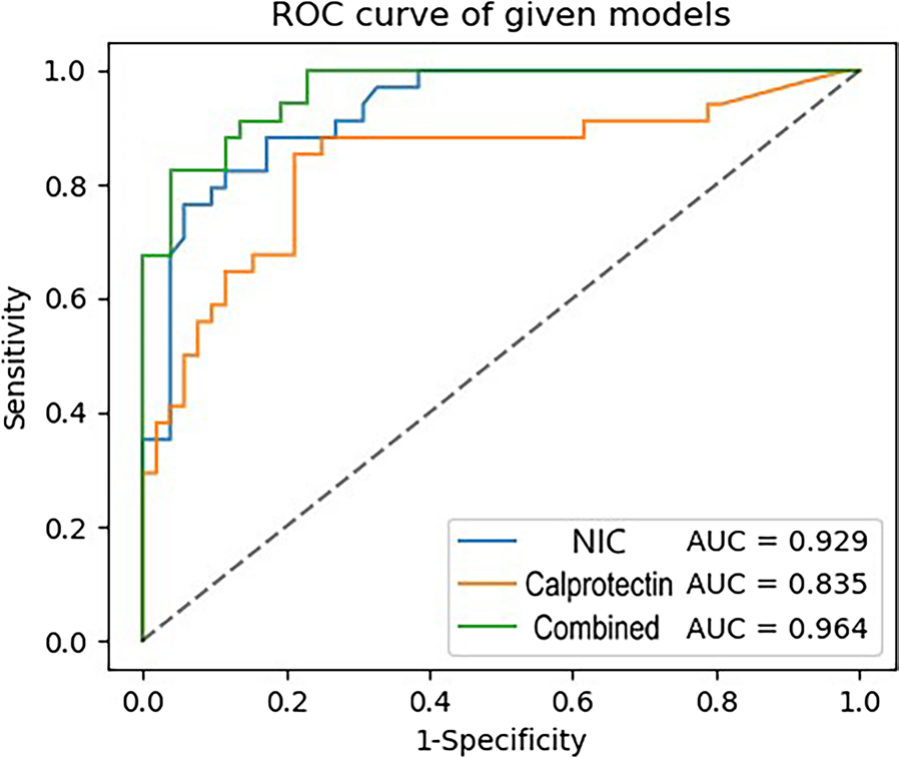
The receiver operating characteristic curve of the NIC, calprotectin, and combined model for the diagnosis of mucosal healing

Following univariate and multivariate logistic regression evaluation, NIC (*p* < 0.001) and calprotectin (*p* = 0.001) remained as the independent predictors for differentiating MH from non-MH. The combined model was established by combining NIC and calprotectin. The AUC of the combined model for distinguishing MH from non-MH in CD patients, which was based on the NIC and calprotectin, was 0.964 (95% CI 0.935–0.987). Table 4 summarizes the discriminatory efficacies of the diagnostic combined model. The ROC curves of the NIC, calprotectin, and combined models are shown in Fig. 5.

**Table 4:** Performance of the NIC, calprotectin and combined models for predicting MH

	AUC (95% CI)	SEN	SPE	ACC
Different models				
NIC	0.929 (0.883–0.967)	0.853	0.827	0.837
Calprotectin	0.835 (0.751–0.915)	0.706	0.788	0.756
Combined model	0.964 (0.935–0.987)	0.794	0.962	0.895

*NIC* Normalized iodine concentration, *AUC* Area under the curve, *SEN* Sensitivity, *SPE* Specificity, *ACC* Accuracy, *95% CI* 95% confidence interval

## Discussion

In the present study, we assessed the use of iodine concentration measurement of the thickened wall of the terminal ileal or colonic segment in DECTE to evaluate endoscopic MH in CD patients and yielded a high AUC of 0.929, indicating a good prediction value for MH in CD patients using iodine concentration measurement in DECTE. To the best of our knowledge, this is the first study to demonstrate the clinical utility of the NIC to predict MH in CD patients, which may provide more information for clinical decision-making.

CTE or MRE findings have been used to assess endoscopic remission. Kim et al. [12] discussed the clinical implications of the CTE findings after endoscopic complete remission of CD in individuals treated with biological treatments. Their results showed that some of the bowel segments that have achieved MH endoscopically continue to show wall thickening of the bowel wall on CTE images. It has been reported that radiologic complete remission (CR) has an edge over endoscopic CR in terms of predicting both clinical and biochemical results in CD patients [13]. TH and radiologic CR have similar definitions, and both indicate complete disappearance of active inflammatory symptoms (< 3 mm) in both mural and extramural areas on cross-sectional images such as CTE or MRE. Of the 94 patients included in our study, 8 patients achieved TH. We found that patients with TH had lower HBI and CRP than those with non-TH, in addition to a return to normal bowel wall thickness. This suggested that patients with TH had better relief in terms of clinical symptoms and laboratory indicators. Our study showed that CTE images can be used to assess TH of CD after IFX treatment, which indicates a fairly favorable prognosis [1, 23].

Iodine concentration measurement in DECTE provides indirect information about blood flow by quantifying the estimated contrast material distribution across the diseased bowel wall at a specific instant in time, and the value of virtual monoenergetic images for the detection of CD has been the subject of numerous reports [22, 24, 25]. Researchers [17] examined qualitative CT features and iodine concentration for the diseased bowel in DECTE images in an effort to correlate quantitative CT characteristics utilizing DECTE with the Crohn's disease activity index (CDAI). The results demonstrated that the iodine concentration measured on the iodine map in DECTE was the only independent variable associated with CDAI. Dane et al. [16] found that iodine concentration from DECTE can be employed as a radiologic measure of histopathologic disease activity in CD. MRE can well reflect the lesion activity and prognosis of CD by means of elastic imaging and changes of different sequence signals. However, MRE examination is time-consuming and expensive. The above articles have shown that the NIC of DECTE is better able to assess Crohn's disease activity and histologic active inflammation grades, and CTE is simple, inexpensive, and suitable for clinical wide development. In clinical practice, we can use NIC to detect MH in patients when they cannot tolerate endoscopy or have intestinal strictures, fistulas, or perforations that prevent them from completing colonoscopy, which also complements endoscopy. Our results demonstrated a relatively high accuracy of 0.837, indicating a good predictive value for MH in CD patients.

In most CD studies, the MH rate for IFX ranged from 30 to 60% [3, 26]. Of the 94 patients included in our study, IFX treatment has 45% efficiency for achieving MH. Following univariate and multivariate logistic regression evaluation, NIC (*p* < 0.001) and calprotectin (*p* = 0.001) remained as the independent predictors for differentiating MH from non-MH. The AUC of the combined model for distinguishing MH from non-MH in CD patients, which was based on the NIC and calprotectin, was 0.964 (95% CI 0.935–0.987). Calprotectin has been an important biomarker for the clinical assessment of MH [20, 27]. We observed that the calprotectin level was significantly lower in the MH group (78.8 ug/g) than in the non-MH group (931.32 ug/g). The findings of our research are consistent with those of previous publications [28–30].

Our study had several limitations. First, we used a manual method to place the ROI on the thickened wall in the area of the highest iodine concentration. Due to the thinness of the transmural healed bowel wall, the ROI was difficult to place accurately and we did not measure the NIC for TH. To increase the efficacy of iodine quantification, automated segmentation and measurement for diseased and transmural healed bowels should be further improved. Second, as a specific DECT scanner (GE Spectrum CT) was used, the results of the study may need to be validated on other DECT machines. Third, MRE has been demonstrated to be a valuable technique to evaluate MH, further comparative studies with MRE are necessary. Finally, there was a relatively small number of patients and a lack of baseline DECTE. Our future studies will focus on collecting DECTE before and after different drugs treatment in CD patients to expand the applicability of the study.

## Conclusion

The DECTE-based assessment of the NIC has good performance in predicting MH in patients with CD. Iodine concentration measurement from DECTE can be used as a radiologic marker of MH in CD patients.

## Data Availability

The data underlying this article cannot be shared publicly due to the privacy of individuals that participated in the study. The data will be shared on reasonable request to the corresponding author.

## Abbreviations

AUC: Area under the ROC curve
CD: Crohn’s disease
CPR: C-reactive protein
CR: Complete remission
CDAI: Crohn’s disease activity index
DECTE: Dual-energy CT enterography
IFX: Infliximab
MH: Mucosal healing
MRE: Magnetic resonance enterography
NIC: Normalized iodine concentration
ROI: Region of interest
ROC: Receiver operating characteristic
SES-CD: Simple endoscopic score for Crohn’s disease
TH: Transmural healing
WBC: White blood cell
